# Cyst of Septum Pellucidum in mental disorders: Schizophrenia and Mental Retardation: Two case reports

**DOI:** 10.1192/j.eurpsy.2023.1953

**Published:** 2023-07-19

**Authors:** M. Belak

**Affiliations:** pedopsychiatric, psychiatric hospital Arrazi, salé, Morocco

## Abstract

**Introduction:**

A septum pellucidum cyst is defined as a fluid collection between the lateral ventricles whose walls have a lateral curvature and are separated by 10 mm or more . Most of these cysts are benign and their clinical significance should be considered as a neurodevelopmental anomaly that may contribute to neuropsychiatric abnormalities . It is often of incidental finding, of little clinical significance. However, an association between this developmental anomaly and a mental disorder, such as schizophrenia and/or intellectual disability, has been reported

**Objectives:**

The objective of this study is to discuss the relationship between the septum pellucidum cyst and mental disorders, especially schizophrenia and intellectual disability.

**Methods:**

We report in this study two clinical cases, diagnosed with schizophrenia comorbid with intellectual disability and in whom brain imaging has objectified a cyst of the septum pellucidum

**Results:**

multiple cases reports of patients with Schizophrenia and/or mental retardation revealed, on brain imaging, significant abnormalities in midline brain regions such as Septum Pellucidum. It is suggested that CSP, particularly if large, should be considered a developmental anomaly that may contribute to neuropsychiatric abnormalities.

**Conclusions:**

Whether the CSP may serve as a risk factor for psychosis or is only a reflection of neuroanatomical changes in individuals with chronic psychotic disorders remains ambiguous. More studies and case reports will be needed to establish the veritable association of CSP and neuropsychiatric disorders in the future, and perhaps to acknowledge the CSP as an early marker and predictor of psychosis.

**Disclosure of Interest:**

None Declared

